# Comprehensive Proteomic Analysis of Dysferlinopathy Unveiling Molecular Mechanisms and Biomarkers Linked to Pathological Progression

**DOI:** 10.1111/cns.70065

**Published:** 2024-09-30

**Authors:** Di Wang, Xin‐yi Liu, Qi‐Fang He, Fu‐ze Zheng, Long Chen, Ying Zheng, Ming‐hui Zeng, Yu‐hua Lin, Xin Lin, Hai‐zhu Chen, Min‐ting Lin, Ning Wang, Zhi‐qiang Wang, Feng Lin

**Affiliations:** ^1^ Department of Molecular Pathology Clinical Oncology School of Fujian Medical University, Fujian Cancer Hospital Fuzhou China; ^2^ Center for Bioinformatics, National Infrastructures for Translational Medicine Institute of Clinical Medicine and Peking Union Medical College Hospital, Chinese Academy of Medical Sciences and Peking Union Medical College Beijing China; ^3^ Department of Neurology and Institute of Neurology of First Affiliated Hospital, Institute of Neuroscience, and Fujian Key Laboratory of Molecular Neurology Fujian Medical University Fuzhou China; ^4^ Department of Neurology, National Regional Medical Center, Binhai Campus of the First Affiliated Hospital Fujian Medical University Fuzhou China

**Keywords:** annexins, dysferlinopathy, inflammation, pathological progression, potential biomarkers, proteomics, therapeutic targets

## Abstract

**Aims:**

Previous proteomics studies in dysferlinopathy muscle have been limited in scope, often utilizing 2D‐electrophoresis and yielding only a small number of differential expression calls. To address this gap, this study aimed to employ high‐resolution proteomics to explore the proteomic landscapes of dysferlinopathy and analyze the correlation between muscle pathological changes and alterations in protein expression in muscle biopsies.

**Methods:**

We conducted a comprehensive approach to investigate the proteomic profile and disease‐associated changes in the muscle tissue proteome from 15 patients with dysferlinopathy, exhibiting varying degrees of dystrophic pathology, alongside age‐matched controls. Our methodology encompasses tandem mass tag (TMT)‐labeled liquid chromatography‐mass spectrometry (LC–MS/MS)‐based proteomics, protein–protein interaction (PPI) network analysis, weighted gene co‐expression network analysis, and differential expression analysis. Subsequently, we examined the correlation between the expression of key proteins and the clinical characteristics of the patients to identify pathogenic targets associated with DYSF mutations in dysferlinopathy.

**Results:**

A total of 1600 differentially expressed proteins were identified, with 1321 showing high expression levels and 279 expressed at lower levels. Our investigation yields a molecular profile delineating the altered protein networks in dysferlinopathy‐afflicted skeletal muscle, uncovering dysregulation across numerous cellular pathways and molecular processes, including mRNA metabolic processes, regulated exocytosis, immune response, muscle system processes, energy metabolic processes, and calcium transmembrane transport. Moreover, we observe significant associations between the protein expression of ANXA1, ANXA2, ANXA4, ANXA5, LMNA, PYGM, and the extent of histopathologic changes in muscle biopsies from patients with dysferlinopathy, validated through immunoblotting and immunofluorescence assays.

**Conclusions:**

Through the aggregation of expression data from dysferlinopathy‐impacted muscles exhibiting a range of pathological alterations, we identified multiple key proteins associated with the dystrophic pathology of patients with dysferlinopathy. These findings provide novel insights into the pathogenesis of dysferlinopathy and propose promising targets for future therapeutic endeavors.

## Graphical abstract

The current study is the first to utilize tandem mass tag (TMT)‐labeled liquid chromatography‐mass spectrometry (LC–MS/MS)‐based proteomics in dysferlinopathy research. The study aimed to explore the proteomic landscape and disease‐related alterations in the muscle tissue proteome of 15 patients with dysferlinopathy with varying degrees of dystrophic pathology, alongside age‐matched controls. Through the analysis of expression data from dysferlinopathy‐affected muscles exhibiting diverse pathological changes, we identified several key proteins linked to the dystrophic pathology observed in patients with dysferlinopathy. These findings provide new insights into the pathogenesis of dysferlinopathy and suggest promising targets for future therapeutic interventions.

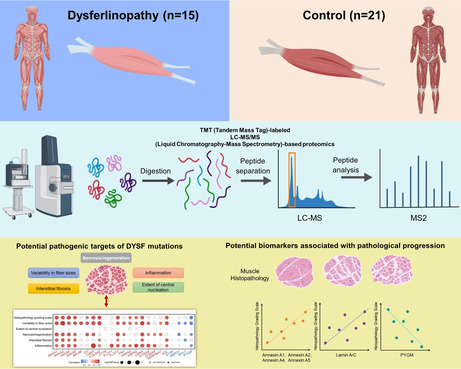

## 1 Introduction

Dysferlinopathies, recessively inherited muscular dystrophies, stem from mutations in the dysferlin gene (MIM#603009, GenBank NM_003494.2) [1, 2]. These mutations lead to diminished or lacking dysferlin protein levels in skeletal muscles, resulting in compromised membrane integrity and hindered wound healing [3]. These conditions primarily present as two major phenotypes: Miyoshi myopathy (MM) and limb‐girdle muscular dystrophy (LGMD) R2 [4].

Dysferlin, a 237‐kDa transmembrane Ca^2+^‐binding protein, consists of seven C2 domains (C2A–C2G), three Fer domains, and two inner DysF domains. Its C2 domains interact with varying affinities for Ca^2+^ and phospholipids, facilitating interactions with multiple protein complexes [5, 6]. In humans, dysferlin is predominantly located in skeletal and cardiac muscles. Its primary functions include fusing intracellular vesicles, anchoring them to the sarcolemma of muscle fibers, and aiding their transportation to sites of membrane disruption [7, 8]. Dysferlin collaborates with proteins such as annexins, caveolin‐3 (Cav3), and mitsugumin 53 (MG53) to repair damaged membranes and restore cell membrane integrity following skeletal muscle injury [9, 10, 11]. Annexins, belonging to a superfamily of calcium‐independent phospholipid‐binding proteins, play critical roles in intracellular calcium homeostasis, vesicle trafficking, and membrane traffic and repair [12, 13]. Both annexins A1 and A2 associate with dysferlin, promoting intracellular vesicle aggregation and fusion in a Ca^2+^‐dependent manner [14]. Dysferlin deficiency disrupts the vesicle fusion process. Furthermore, dysferlin colocalizes with the dihydropyridine receptor and AHNAK, contributing to Ca^2+^ homeostasis and the maintenance of the T‐tubule system [15, 16]. Dysferlin's association with α‐tubulin and microtubules, facilitated by its C2A and C2B domains, is involved in vesicle trafficking. It also interacts with SNARE proteins such as syntaxin 4 and SNAP‐23, implicating it in cytokine secretion and lysosome exocytosis [17, 18].

Although previous studies have primarily focused on exploring the functional role of dysferlin in sarcolemma membrane repair, numerous aspects of dysferlinopathy's pathomechanism remain elusive. Therefore, it is important to elucidate the intricate molecular mechanisms underlying dysferlinopathy, particularly at the proteome level. Early investigations into dysferlinopathy muscle employed 2D‐electrophoresis to identify differentially abundant proteins [19, 20, 21]. However, these studies yielded limited results, with only a small number of differential expression calls. Other methodologies, such as label‐free approaches, have identified additional differentially expressed proteins in dystrophic muscle. Nevertheless, these studies are constrained by their low proteomic coverage. Analyzing the complete proteome in fibrous tissues like skeletal muscle poses challenges because of the significant abundance of key structural proteins, such as myosins and actins [22]. Peptides generated from these proteins obscure signals from less abundant proteins, thus limiting the attainable depth of proteome coverage attainable. To enhance analytical depth, we quantified the expression of over 1500 proteins in dysferlinopathy patient muscle using high‐resolution sample pre‐fractionation based on narrow‐range isoelectric focusing of peptides.

Muscle biopsies from patients with dysferlinopathy reveal nonspecific dystrophic changes, including variations in the size of muscle fibers, increased internal nuclei, and degenerating or regenerating fibers. As the disease progresses, there is also an increase in interstitial fibrosis. Inflammatory infiltrates may occur and can even be prominent in dysferlinopathy; however, the pattern differs from that observed in inflammatory myopathies [23, 24]. Pathology grading systems are instrumental in quantifying muscle pathological findings, offering a crucial measure of disease progression necessary for future clinical trials. Currently, the correlation between muscle pathological changes and alterations in protein expression in muscle biopsies remains elusive. Integrating proteomic expression data with muscle pathology changes could significantly enhance our comprehension of the pathophysiological mechanisms driving dysferlinopathy.

Until now, limited high‐resolution proteomics investigations have been conducted in dysferlinopathy muscle, with none assessing global changes in protein expression in patients with dysferlinopathy correlated with different muscle pathology grades. In this study, we conducted MS‐based proteomic profiling in 15 patients with dysferlinopathy exhibiting varying degrees of dystrophic pathology, comparing them with 21 controls. Our findings demonstrate that this state‐of‐the‐art proteomic technology has unveiled previously unidentified pathological pathways, thereby broadening our understanding of the molecular pathogenesis of dysferlinopathy. These results offer promising avenues for potential therapeutic targets against the disease.

## 2 Methods

### 2.1 TMT‐Labeled LC–MS/MS‐Based Proteomics

#### 2.1.1 Sample Preparation for Mass Spectrometry

Muscle samples were subjected to protein extraction and mixed with a lysis buffer consisting of 8 M urea and 1% protease inhibitor cocktail. The mixture underwent three rounds of sonication on ice using a high‐intensity ultrasonic processor (Scientz). Any remaining solid particles were removed through centrifugation at 12,000 *g* at 4°C for 10 min. The resulting supernatant was collected, and the protein concentration was determined using a BCA kit as per manufacturer guidelines.

To prepare for trypsin digestion, the solution was reduced using 5 mM dithiothreitol and kept at 56°C for 30 min. Subsequently, it was alkylated with 11 mM iodoacetamide in the dark at room temperature for 15 min. The protein sample was then diluted by adding 100 mM triethylammonium bicarbonate (TEAB) until the urea concentration dropped below 2 M. Finally, the first trypsin digestion occurred overnight at a trypsin‐to‐protein mass ratio of 1:50, and the second digestion took place for 4 h at a trypsin‐to‐protein mass ratio of 1:100.

#### 2.1.2 TMT‐Labeled and HPLC Fractionation

The peptides were subjected to a desalting process using a Strata X C18 SPE column (Phenomenex) and subsequently dried under vacuum. The peptides were then reconstituted in 0.5 M TEAB and further processed in accordance with the instructions provided by the tandem mass tag (TMT) kit. Tryptic peptides were separated into fractions using Thermo Betasil C18 column (5 μm particles, 10 mm ID, and 250 mm length) and high pH reverse‐phase HPLC.

#### 2.1.3 LC–MS/MS Analysis

The tryptic peptides were solubilized in 0.1% formic acid (as solvent A) and then directly applied to a custom‐made reversed‐phase analytical column (15 cm length, 75 μm i.d.). The gradient consisted of a transition from 6% to 23% solvent B (0.1% formic acid in 98% acetonitrile) within 26 min, followed by an increase from 23% to 35% in 8 min, and a final rise to 80% in 3 min. It maintained an 80% composition for the last 3 min, all at a consistent flow rate of 400 nL/min on an EASY‐nLC 1000 UPLC system. The peptides underwent NSI source followed by tandem mass spectrometry (MS/MS) using the Q ExactiveTM Plus instrument by Thermo, which was connected to the UPLC system. The survey scan was performed in the Orbitrap at a resolution of 70,000 from 350 to 1800 m/z, with maximum IT set to 100 ms. Peptides were chosen for MS/MS with an NCE setting of 28, and the resulting fragments were identified in the Orbitrap at a resolution of 17,500. A data‐dependent process that alternated between 1 MS scan followed by 20 MS/MS scans, with a dynamic exclusion of 15.0 s.

#### 2.1.4 Peptide and Protein Identification

The resulting MS/MS data were analyzed using the MaxQuant search engine (v.1.5.2.8). The tandem mass spectra were compared with the human Uniprot database, which was combined with a reverse decoy database for the matching process. Trypsin/P was designated as the cleavage enzyme with allowance for up to four missed cleavages. The precursor ion mass tolerance was set to 20 ppm in the initial search and 5 ppm in the subsequent search, whereas the fragment ion mass tolerance was established at 0.02 Da. Carbamidomethyl modification on Cysteine was set as a fixed modification, and modifications such as acetylation and oxidation on Methionine were defined as variable modifications.

### 2.2 DYSF Mutation and Expression

We obtained the normalized RNA expression levels of DYSF in 55 normal tissue types from the Human Protein Atlas (RNA consensus tissue gene data) [25]. The reported mutation entries of DYSF in LGMD were obtained from the Human Gene Mutation Database [26]. We obtained the protein domains of DYSF from the Pfam database. We utilized Gene ORGANizer to query body parts that are affected by DYSF [27].

### 2.3 Construction and Analysis of Protein–Protein Interaction Networks

We downloaded all the proteins interacting with DYSF and their interaction relationships from STRING [28]. Subsequently, we constructed the protein–protein interaction (PPI) network of these proteins in Cytoscape [29]. We then employed the MCODE plugin to detect densely connected modules in the PPI network [30].

### 2.4 Functional Enrichment Analysis

Functional enrichment analysis was conducted utilizing the clusterprofiler package [31]. The false discovery rate (FDR) was calculated by adjusting the *p* value using the Benjamini–Hochberg method, with an FDR threshold of ≤ 0.05 deemed as statistically significant.

### 2.5 Weighted Gene Co‐Expression Network Analysis

We conducted weighted gene co‐expression network analysis (WGCNA) using the WGCNA package to identify protein modules associated with DYSF expression in normal muscle tissue [32]. We set the *R* ^2^ cutoff to 0.9 and determined the power to be 6 using the pickSoftThreshold function to construct signed co‐expression networks. Modules within the network were identified through hierarchical clustering. Subsequently, we conducted Pearson correlation analysis between the module eigengene (ME) and DYSF protein expression, considering a *p* value of ≤ 0.05 to be significant.

### 2.6 Muscle Immunofluorescence and Western Blot Analysis

Routine histological analysis was conducted on 8‐μm‐thick frozen cross‐sections. The pathology grade utilizes an ordinal scale to assess various morphological characteristics of dysferlinopathy muscle biopsies, assigning a score that ranges from 0 to 3 (0 = normal; 0.5 and 1 = mild; 1.5 and 2 = moderate; and 2.5 and 3 = severe). The pathology grade is calculated as the sum of the scores across five categories: variability in fiber size, extent of central nucleation, necrosis/regeneration, interstitial fibrosis, and inflammatory infiltrates [33]. The scores are independently determined by two neuromuscular pathologists on the basis of their impressions of the sample pathology.

Frozen muscle biopsies were sectioned into 7 μm‐thick slices, placed on slides, and allowed to air‐dry for 20 min at room temperature. For immunofluorescence analysis, the sections were rinsed with PBS, fixed in 4% PFA (paraformaldehyde) for 15 min, permeabilized with 0.1% Triton X‐100, and subsequently blocked with bovine serum albumin (Beyotime). The sections were incubated overnight at 4°C with primary antibodies, including anti‐annexin A1 (ab214486, 1:250; Abcam), anti‐annexin A2 (ab178677, 1:200; Abcam), anti‐annexin A4 (ab256456, 1:200; Abcam), anti‐annexin A5 (ab108194, 1:250; Abcam), anti‐Lamin A/C (#MA3‐1000, 1:250; ThermoScientific), dystrophin (NCLdys1, 1:100; ThermoScientific), and CD31 (66,065‐2‐Ig, 1:200; Proteintech). After three PBS washes (5 min each), the sections were incubated with secondary antibodies at room temperature for 1 h donkey anti‐rabbit IgG (H + L) conjugated with Alexa Fluor 488, A‐21206, or goat anti‐mouse IgG (H + L) conjugated with Alexa Fluor 594 (A32742; 1:500; Invitrogen). Some coverslips were counterstained with 4′,6‐diamidino‐2‐phenylindole (DAPI, ab104139; Abcam), and the sections were visualized using a Zeiss LSM 880 confocal microscope. Muscle specimen from patients with dysferlinopathy with different muscle pathologies and control cases. Immunoblot analysis was performed with the primary antibodies: anti‐Flot2(ab96507, 1:6000; Abcam), anti‐Lamin A/C (ab108595, 1:1000; Abcam), PYGM (ab231963, 1:5000; Abcam), and anti‐GAPDH (AF0006, 1:6000; Beyotime Biotechnology). Protein intensities from immunoblots and fluorescence intensities from immunofluorescence staining were quantified using ImageJ.

### 2.7 Differential Protein Analysis

Differential proteins between LGMD muscle tissue and normal muscle tissue were identified using limma, and those with a FDR ≤ 0.05 were considered significantly differentially expressed [34].

### 2.8 Statistical Analysis

All statistical analyses were conducted using R (version 4.1.1). Pearson correlation analysis was executed using the cor.test function. The Gaussian distribution of the data for statistical analysis was assessed using the Shapiro–Wilk test. For normally distributed data, comparisons between two groups were performed using a two‐tailed *t*‐test. For multiple comparisons, statistical significance was assessed using a one‐way ANOVA followed by Dunnett's correction. When the data were non‐Gaussian distributed, the Mann–Whitney *U*‐test was used for comparing two groups. A *p* value of < 0.05 was considered statistically significant.

## 3 Results

### 3.1 Patients and Clinical Data

In this study, 15 Chinese patients participated, comprising nine females and six males. Among these patients, four presented with distal MM, two exhibited the proximo‐distal (PD) phenotype, and nine displayed the LGMD phenotype (Table 1) [35]. The onset of symptoms occurred between the ages of 15 and 42, with an average onset age of 23.6 years. Their muscle biopsies were conducted at ages ranging from 18 to 45 years (average age: 29.7), occurring at various intervals following the onset, spanning from 1 to 18 years, with an average disease duration of 6.1 years. Subsequently, the muscle specimens underwent a series of histochemical tests to categorize them based on their muscle pathological severity score (Table 1).

**TABLE 1:** Clinical and molecular data of 15 patients with dysferlinopathy.

Patient	Sex/Age of onset	Biopsy age (years)	Muscle biopsy	Clinical phenotypes	CK level (IU/L)	Nucleotide change	Mutation effect	Histopathology Grading Scale	Variability in fiber sizes	Extent of central nucleation	Necrosis/Regeneration	Interstitial fibrosis	Inflammation
1	M/15	25	Biceps brachii	MM	6943	Hom c.937 + 1G > A	Abl.spl	5	1	1	1	1	1
2	F/19	24	Tibialis anterior	LGMD2B	10,530	Hom c.1667T > C	p.L556P	8.5	1.5	2.5	1.5	1.5	1.5
3	F/17	18	Tibialis anterior	MM	3006	c.586_587insC c.856‐1G > T	p.H196Pfs*15 Abl.spl	4	1	0.5	1	1	0.5
4	F/24	32	Vastus lateralis	PD	5407	Hom c.797‐798delTT	p.F267Lfs*5	8.5	2	1	2	2	1.5
5	F/16	19	Biceps brachii	LGMD2B	19,611	c.89‐2A > G c.2810+1G > A	Abl.spl Abl.spl	2	0.5	0	1	0.5	0
6	M/31	36	Tibialis anterior	LGMD2B	16,866	Hom c .1667T > C	p.L556P	7.5	2	1.5	1.5	1	1.5
7	F/21	24	Tibialis anterior	MM	7343	Hom c.797‐798delTT	p.F267Lfs*5	8.5	2.5	0	2	2.5	1.5
8	M/34	44	Biceps brachii	LGMD2B	8343	c.1667T > C c.6124C > T	p.L556P p. R2042C	6.5	2	1	1.5	1	1
9	M/26	27	Vastus lateralis	MM	4328	c.5694dupT c.937+1G > A	p.E1899* Abl.spl	10.5	2.5	1	2.5	2.5	2
10	F/20	21	Tibialis anterior	PD	6551	c.5836C > T c.3102C > G	p.Q1946* p.Y1034*	8.5	2	2	1.5	1.5	1.5
11	F/18	35	Biceps brachii	LGMD2B	—	c.3103_3104delinsAGATCG c.836A > T	p.T1035Afs*80 Abl.spl	8	2.5	0	2	2	1.5
12	M/19	20	Biceps brachii	LGMD2B	31,740	c.6217A > G c.5350C > T	p.M2073V p.Q1784*	6.5	1.5	0.5	2	0.5	2
13	F/42	45	Tibialis anterior	LGMD2B	1868	Hom c .1667T > C	p.L556P	4.5	1	1	1	1	0.5
14	M/19	37	Biceps brachii	LGMD2B	1808	Hom c .1667T > C	p.L556P	6	1.5	1	1.5	1	1
15	F/33	39	Biceps brachii	LGMD2B	4164	c.1667T > C c.6124C > T	p.L556P p.R2042C	8.5	1.5	1	2	3	1

Abbreviations: F, female; LGMD2B, limb‐girdle muscular dystrophy 2B; M, male; MM, Miyoshi myopathy; PD, proximo‐distal.

### 3.2 Functional Analysis of *DYSF*

**FIGURE 1:**
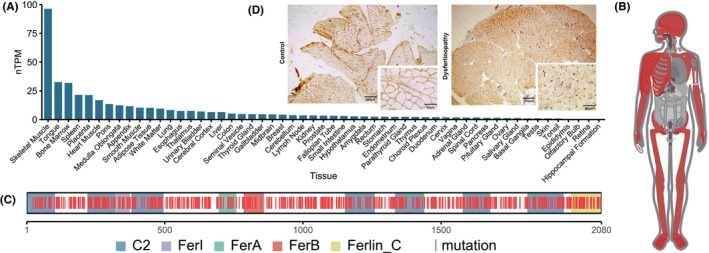
DYSF functions. (A) Bar graph showing RNA expression levels of DYSF in various tissues. nTPM, normalized transcripts per million. (B) Body map showing systems affected by the mutation of DYSF. (C) Schematic showing reported mutation sites of DYSF. C2, C2 domain; FerA, FerA domain; FerB, FerB domain; FerI, FerI domain; Ferlin_C, Ferlin C_terminus. (D) DYSF mutations in dysferlinopathy lead to reduced protein expression in the skeletal sarcolemma.

We initially characterized the function of DYSF by investigating its expression and mutations. Among 55 tissue types, *DYSF* exhibits the highest expression in skeletal muscle at 96.3 normalized transcripts per million (nTPM) (Figure 1A). This specific expression pattern of *DYSF* underscores its pivotal role in skeletal muscle function. As indicated by Gene ORGANizer, *DYSF* influences both the nervous and skeletal muscle systems (Figure 1B). Notably, *DYSF* is a well‐established pathogenic gene associated with dysferlinopathy, and the reported gene mutation sites do not exhibit positional domain specificity (Figure 1C). Mutations in *DYSF* observed in patients with dysferlinopathy result in decreased expression of its protein in skeletal muscle (Figure 1D). These results indicate that DYSF is closely related to the function of skeletal muscle.

### 3.3 DYSF‐Related Protein Interaction Network Analysis

**FIGURE 2:**
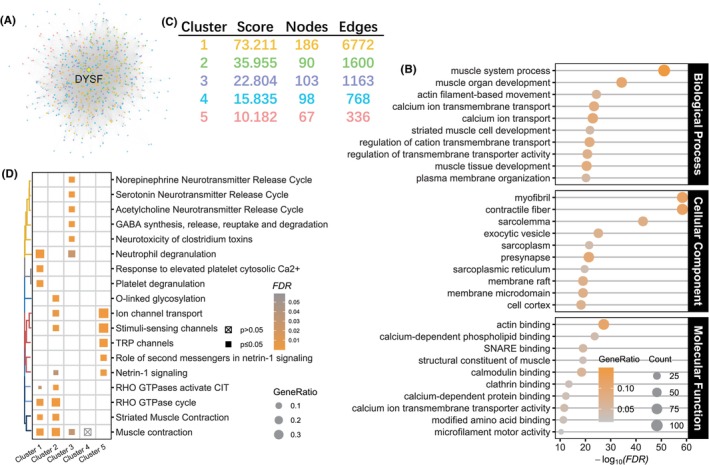
DYSF interactome. (A) Protein–protein interaction network of proteins that interact with DYSF. Each dot represents a protein. Different colors are represented in different clusters assigned using MCODE. (B) Top enriched terms from results of the Gene Ontology (GO) enrichment analysis of proteins that interact with DYSF. (C) Table showing information of five pivotal clusters identified by MCODE. (D) Results of the REACTOME pathway enrichment analysis of proteins in five pivotal clusters.

We identified highly interacting protein clusters related to DYSF from the PPI network. According to STRING, we obtained 744 proteins that interacted with DYSF, resulting in 38,485 interactions (Figure 2A). Subsequently, we conducted the Gene Ontology enrichment analysis to elucidate the functions of these proteins. In the biological process category, the most significantly enriched terms included muscle system process, muscle organ development, actin filament‐based movement, and calcium ion transport. Similarly, in the molecular function category, actin binding, calcium‐dependent phospholipid binding, SNARE binding, structural constituent of muscle, and calmodulin binding emerged as the most significantly enriched terms. Regarding the cellular component category, the terms myofibril, contractile fiber, sarcolemma, exocytic vesicle, and sarcolemma exhibited the highest enrichment (Figure 2B). Furthermore, employing the MCODE algorithm, we identified five pivotal clusters with a MCODE score greater than 10 (Figure 2C). Notably, Cluster 1 displayed the highest score, comprising 186 nodes, including DYSF, and 6772 edges. Subsequent REACTOME pathway enrichment analysis revealed cluster‐specific pathways. Specifically, proteins within Cluster 1 were significantly enriched in pathways such as neutrophil degranulation, response to elevated platelet cytosolic Ca^2+^, platelet degranulation, RHO GTPases activate CIT, RHO GTPase cycle, striated muscle contraction, and muscle contraction (Figure 2D). Thus, we identified protein clusters that highly interact with DYSF and their functions.

### 3.4 DYSF‐Related Co‐Expression Modules

**FIGURE 3:**
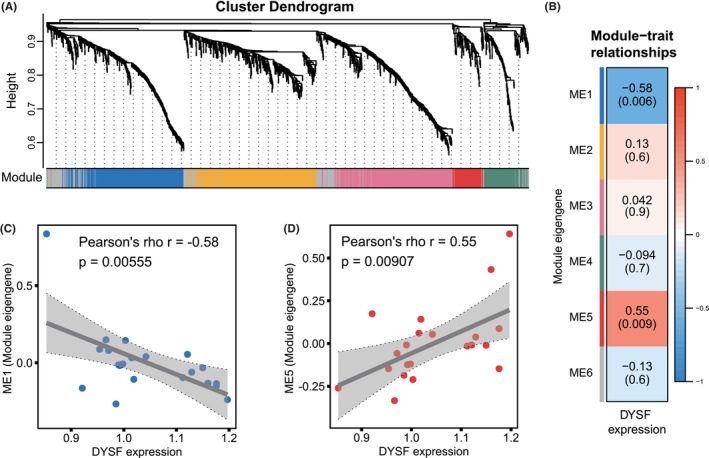
DYSF‐related co‐expression modules. (A) Cluster dendrogram showing the co‐expression modules identified using weighted gene co‐expression network analysis (WGCNA). (B) Heatmap showing the module‐trait relationship (*p* value) for the identified modules in relation to the DYSF expression value. ME, module eigengene. (C) Scatterplot showing the correlation between the ME1 module eigengene and DYSF expression. (D) Scatterplot showing the correlation between the ME5 module eigengene and DYSF expression.

In our study, we utilized WGCNA to pinpoint co‐expression modules linked to DYSF expression in normal skeletal muscle tissues. Among 21 normal skeletal muscle samples, we identified five protein modules showing high correlation (Figure 3A). Subsequently, we conducted correlation analyses between the ME, which serves as a representation of module expressions, and DYSF protein expression (Figure 3B). Notably, the ME1 exhibited a significant negative correlation with DYSF expression (Figure 3C), whereas conversely, the ME5 displayed a significant positive correlation (Figure 3D). In summary, we identified two co‐expression modules associated with DYSF expression in normal skeletal muscle tissues.

### 3.5 Differentially Expressed Proteins in Dysferlinopathy

**FIGURE 4:**
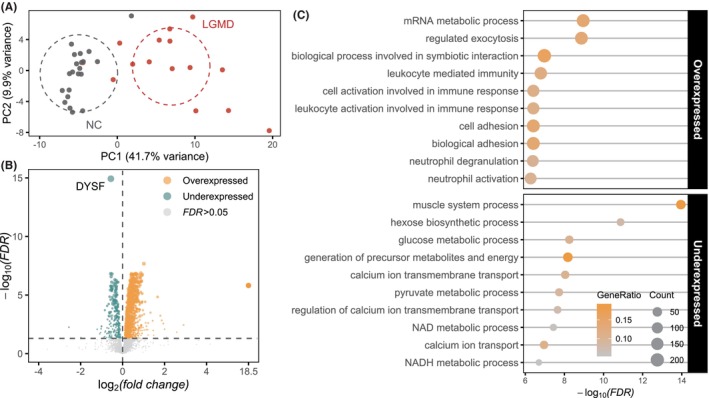
Proteomic differences between limb‐girdle muscular dystrophy (LGMD) and normal tissues. (A) Principal component analysis of proteomics data for LGMD and normal tissues. (B) Volcano plot showing differentially expressed proteins in LGMD and normal tissues. FDR, false discovery rate. (C) Results of the REACTOME pathway enrichment analysis of both overexpressed and under‐expressed proteins.

To characterize abnormal proteins in dysferlinopathy, we conducted differential protein analysis comparing dysferlinopathy and normal tissues. Initially, we confirmed proteomic distinctions between dysferlinopathy and normal tissues using PCA (Figure 4A). Subsequently, we identified 1600 differentially expressed proteins (FDR ≤ 0.05). This comprised 1321 proteins showing high expression and 279 proteins showing low expression (Figure 4B). Our analysis revealed that high‐expressed proteins were notably enriched in several biological processes, including mRNA metabolic process, regulated exocytosis, biological processes involved in symbiotic interaction, leukocyte‐mediated immunity, and cell activation associated with immune response. Conversely, low‐expressed proteins demonstrated significant enrichment in processes related to the muscle system, hexose biosynthesis, glucose metabolic pathways, generation of precursor metabolites and energy, and calcium ion transmembrane transport (Figure 4C). Through this analysis, we identified differentially expressed proteins between dysferlinopathy and normal tissues and characterized their functions.

### 3.6 Identification of Pathogenic Targets of DYSF Mutations

**FIGURE 5:**
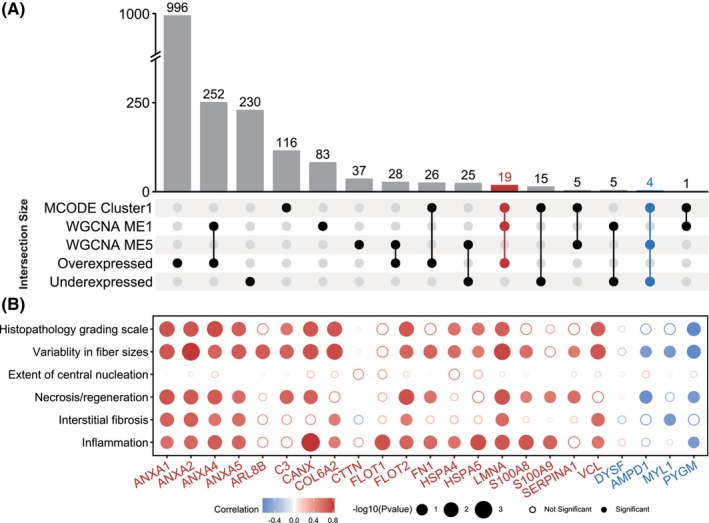
Potential pathogenic targets of DYSF mutations. (A) UpSet plot showing the intersections of multiple protein sets. (B) Bubble plot showing the correlation between protein expression (X‐axis) and clinical characteristics (Y‐axis).

**FIGURE 6:**
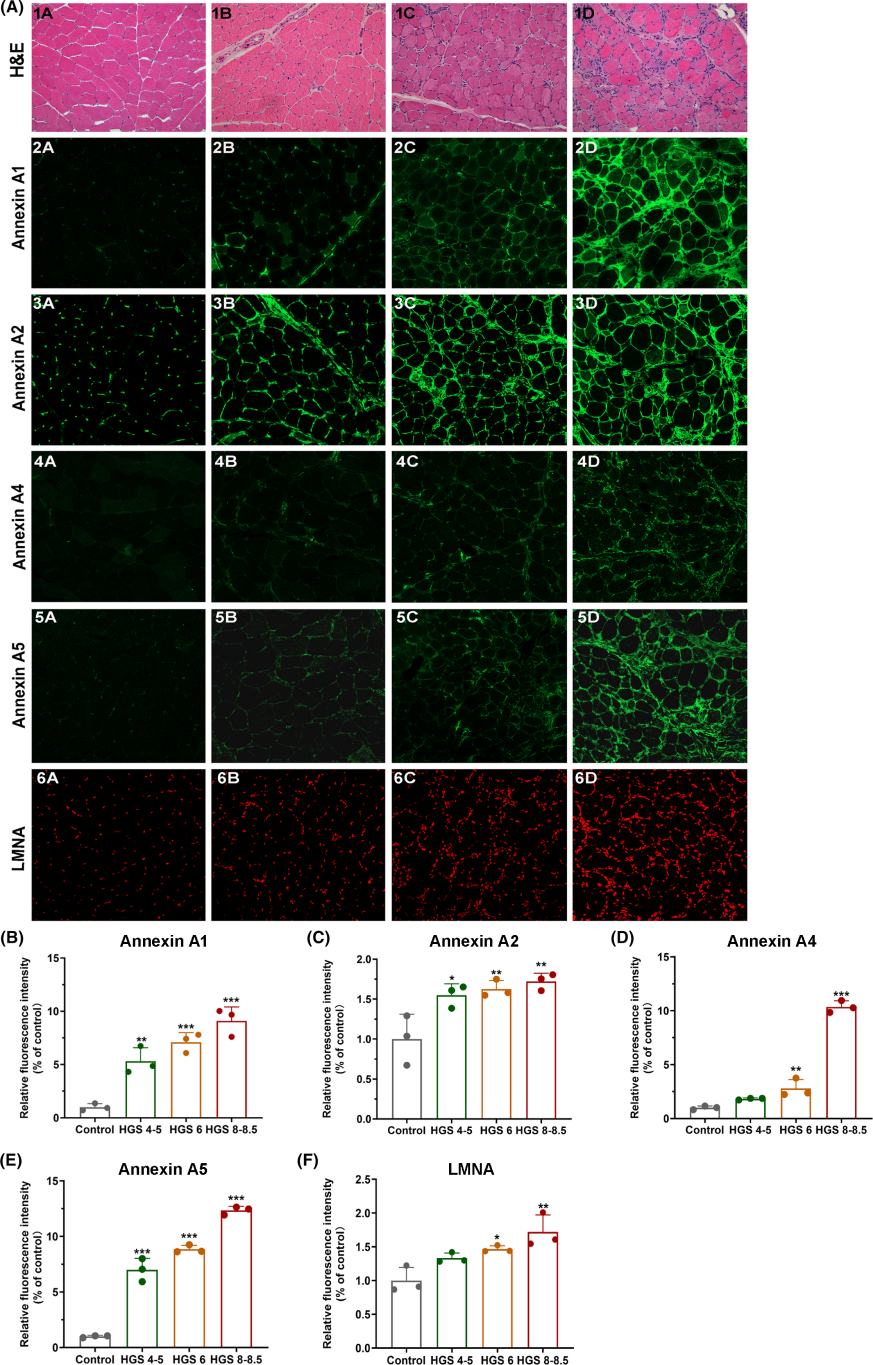
Hematoxylin and eosin (H&E) staining and immunofluorescence analysis of patients with dysferlinopathy with varying degrees of dystrophic pathology. Column 1: muscle sample from a normal control subject, H&E (1A), and minimal or absence of the expression of annexin A1 (2A), annexin A2 (3A), annexin A4 (4A), and annexin A5 (5A) in control muscle. Normal expression of Lamin A/C (6A) in control muscle. Column 2: mild dystrophic changes in patients with dysferlinopathy (Patients 1 and 3, Histopathology Grading Scale of 4–5), H&E in Patient 3 (1B), expression of annexin A1 (2B) in Patient 1, and expression of annexin A2 (3B), annexin A4 (4B), annexin A5 (5B), and Lamin A/C (6B) in Patient 3. Column 3: moderate dystrophic features in patient 14 (Histopathology Grading Scale 6), H&E in Patient 14 (1C), and expression of annexin A1 (2C), annexin A2 (3C), annexin A4 (4C), annexin A5 (5C), and Lamin A/C (6C) in Patient 14. Column 4: Moderate–severe dystrophic changes in dysferlinopathy (Patients 10 and 11, Histopathology Grading Scale 8–8.5), H&E in Patient 10 (1D), expression of annexin A1 (2D), annexin A2 (3D), and Lamin A/C (6D) in Patient 11, and expression of annexin A4 (4D) and annexin A5 (5D) in patient 10. (B–F) Relative fluorescence intensities of annexin A1, annexin A2, annexin A4, annexin A5, and Lamin A/C in controls and patients with different ranks on the Histopathology Grading Scale. Each bar indicates the mean ± SD. *p < 0.05 versus Control, **p < 0.01 versus Control, ***p < 0.005 versus Control.

**FIGURE 7:**
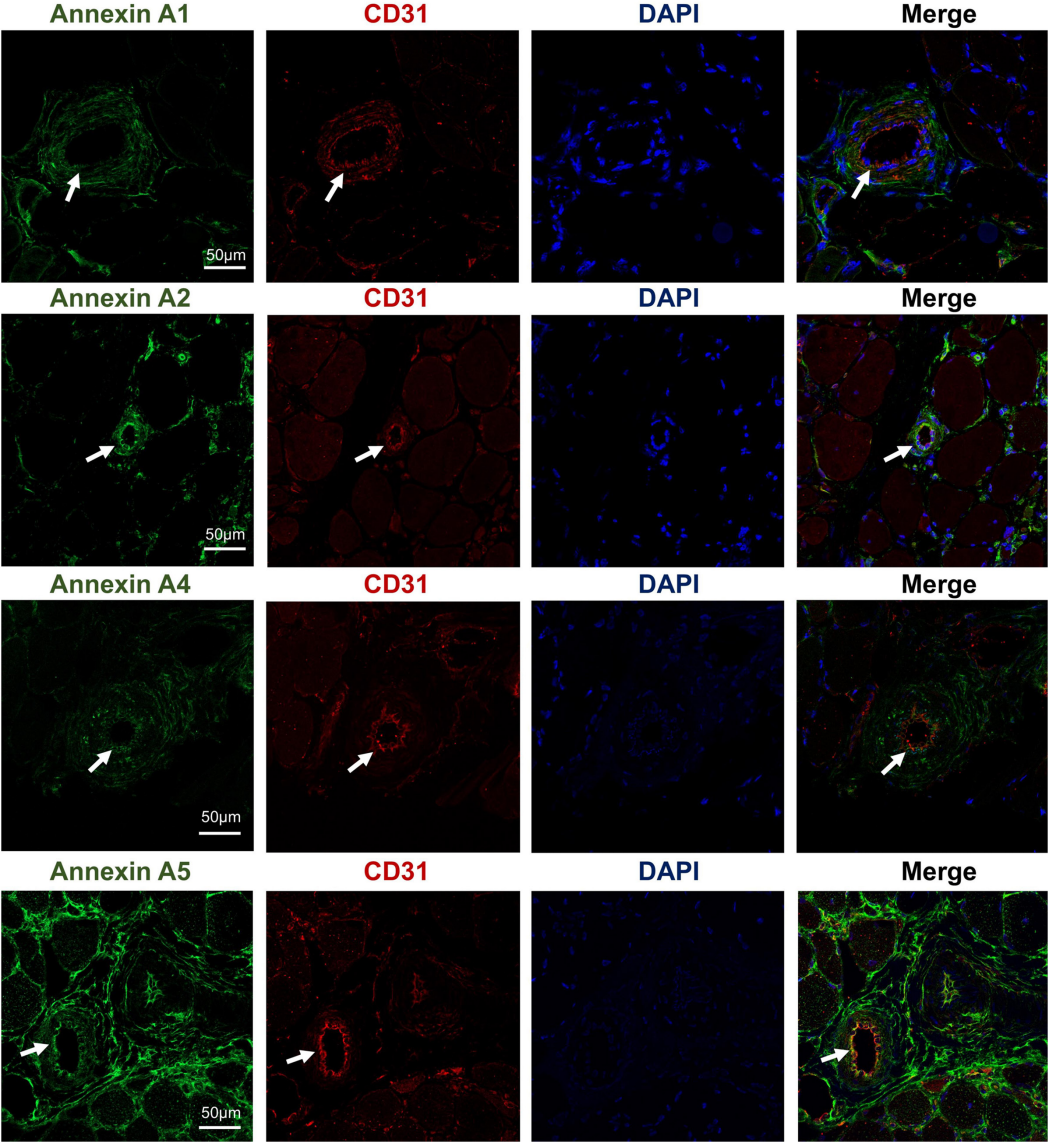
Double‐label immunofluorescence shows that annexin A1, annexin A2, annexin A4, and annexin A5 partially colocalize with CD31, a marker of small vessel endothelium.

**FIGURE 8:**
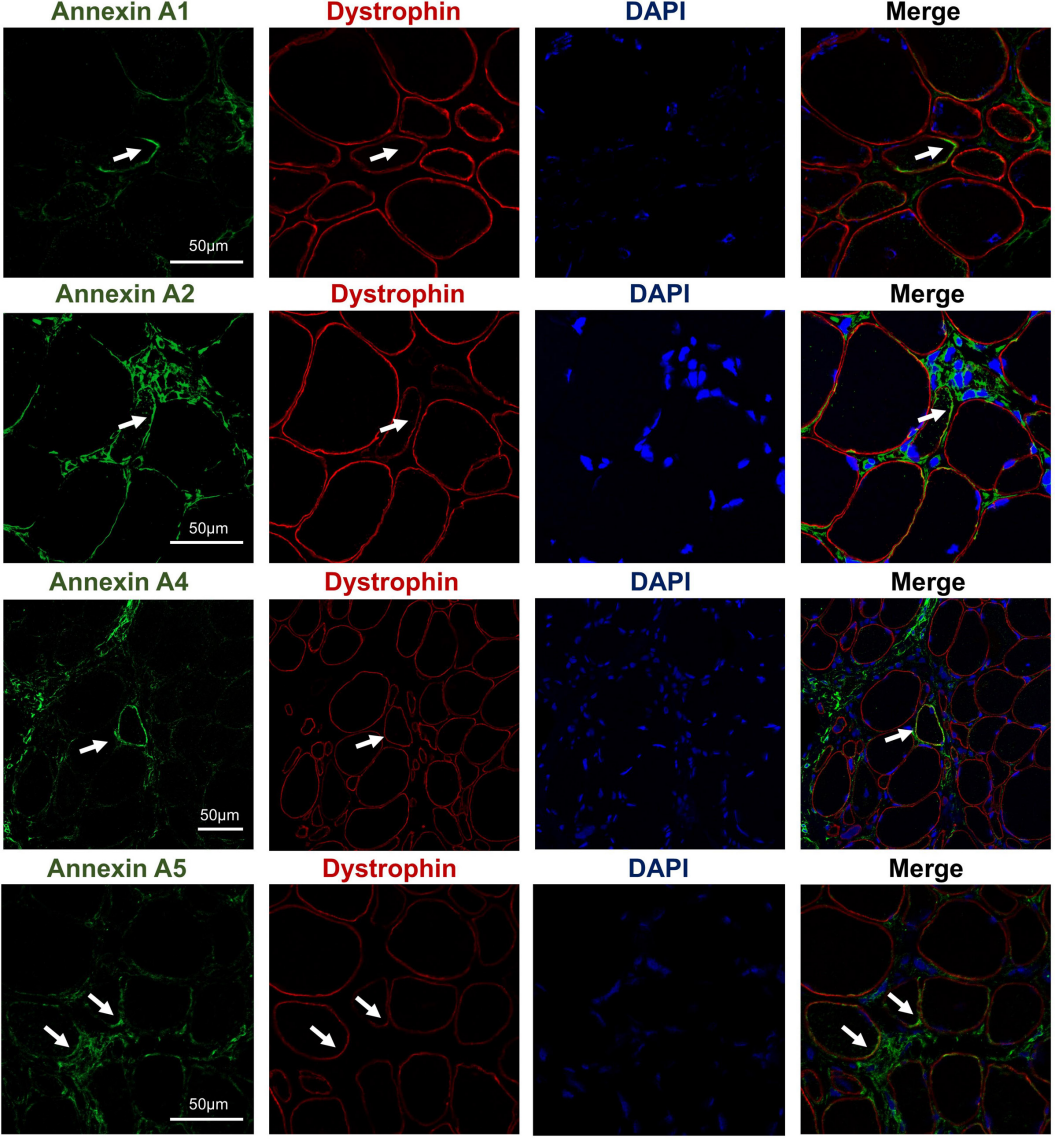
Colocalization of annexin A1, annexin A2, annexin A4, annexin A5, and dystrophin, a marker of the muscle fiber sarcolemma.

**FIGURE 9:**
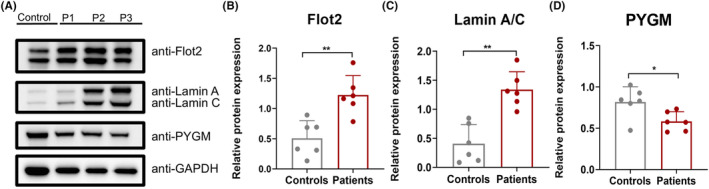
FLOT2, Lamin A/C, and PYGM immunoblot analysis in muscle biopsies from patients with dysferlinopathy with varying degrees of dystrophic pathology. (A) Increased levels of FLOT2 and Lamin A/C, along with decreased levels of PYGM, were clearly detectable in all analyzed patients. (B–D) Quantitative analysis of FLOT2, Lamin A/C, and PYGM level relative to GAPDH. Each bar indicates the mean ± SD. *p < 0.05 versus Control, **p < 0.01 versus Control (*n* = 6 in each group).

Based on the aforementioned results, we investigated the pathogenic targets of DYSF mutations in LGMD. In the upset plot, we identified 19 genes present in both MCODE Cluster 1, WGCNA module 1, and overexpressed genes, whereas four genes were common to both MCODE Cluster 1, WGCNA module 5, and under‐expressed genes (Figure 5A). Subsequently, we conducted correlation analysis between the protein expression of these genes and the clinical characteristics of the patients. Our findings revealed significant positive correlations between ANXA1, ANXA2, ANXA4, ANXA5, FLOT2, and LMNA with parameters such as Histopathology Grading Scale, variability in fiber sizes, necrosis/regeneration, interstitial fibrosis, and inflammation (Figure S1). Conversely, PYGM (glycogen myophosphorylase) showed a significant negative correlation with histopathology grading scale, variability in fiber sizes, necrosis/regeneration, and inflammation (Figure 5B and Figure S1). To verify the expression of these potential biomarkers in dysferlinopathy, we conducted immunofluorescence and immunoblot analyses on patients with varying histopathological grades. The expression of Annexins and Lamin A/C correlated with the degree of the dystrophic process and the severity of muscle histopathology (Figure 6). In control muscles, annexins were primarily expressed weakly in the extracellular matrix. However, in patients' muscles, annexin signals could be visualized not only in the extracellular matrix but also in the small vessel endothelium and sarcolemma (Figures 7 and 8). In immunoblot analyses of LMNA, PYGM, and FLOT2 expression, we observed increased expression of these proteins in all patients compared with control muscles (Figure 9). The densitometric analysis of Lamin A/C and PYGM levels indicates that patients with higher histopathology scores tended to exhibit elevated levels of Lamin A/C, while showing reduced expression of PYGM. In summary, we integrated proteomic analysis results to identify the pathogenic targets of DYSF mutations in LGMD and experimentally validated these findings.

## 4 Discussion

The investigation into proteomic profiles underlying dysferlinopathy holds importance in comprehending the disease's pathophysiology, discerning potential therapeutic targets, and formulating biomarkers for disease progression or clinical response. In this study, we utilized MS‐based profiling to elucidate the proteomic characteristics of dystrophic muscle exhibiting varying pathology grades in dysferlinopathy. Despite encountering technical challenges inherent in skeletal muscle proteomics, we successfully quantified 1600 differentially expressed proteins. This represents the most extensive catalog of measured proteins in dysferlinopathy muscle documented to date. Pathway analysis was executed to unravel the biological significance of differential protein expression. Our findings indicate that dysferlinopathy is marked by the expression of proteins linked to mRNA metabolic processes, regulated exocytosis, immune response, and cell adhesion. In contrast, protein signatures associated with muscle system processes, calcium ion transport, and cellular energy metabolism (including hexose biosynthesis, pyruvate metabolism, glucose metabolism, and NAD/NADH metabolism) were observed to be downregulated in dystrophic muscle. By consolidating expression data from muscles affected by dysferlinopathy with diverse degrees of pathological changes, we identified pivotal proteins implicated in dystrophic pathology. Furthermore, these findings were further validated through immunoblotting and immunofluorescence techniques. Ultimately, we identified multiple key proteins closely associated with the clinical features of patients with dysferlinopathy.

### 4.1 Inflammation Response and Complement Activation in Dysferlinopathy

Dysferlin deficiency within myofibers induces impaired sarcolemma repair, disruptions in calcium balance within T‐tubules, and alterations in the immune response, leading to progressive muscular disorders characterized by limited regenerative capacity, the presence of inflammatory infiltrates, and the development of fibro‐adipogenesis. Although inflammation and immune cell infiltrates are frequently observed in muscle biopsies of individuals with dysferlinopathy, the precise mechanisms by which the immune system contributes to the disease pathology remain not fully understood. Disturbed immune responses, including dysregulation of the inflammasome pathway, alterations in macrophage function, activation of complement systems, the presence of the membrane attack complex on myofibers, and increased levels of oxidative stress, have been revealed through studies in both humans and dysferlin‐deficient mice [36, 37, 38, 39, 40, 41].

Baek et al. [42] highlighted the recruitment of intramuscular inflammatory M1 macrophages in dysferlinopathic muscles, a process that fosters their proliferation and heightens muscle fibers' susceptibility to necrosis and apoptosis. Within dysferlin‐deficient dystrophic muscles, toll‐like receptor (TLR)‐mediated signaling pathways kickstart an inflammatory cascade, activating NF‐κB and prompting the formation of the NLRP3 inflammasome [37, 43]. Inhibition of the innate immune response governed by TLRs has shown promise in diminishing muscle atrophy and enhancing muscle strength in dysferlin‐deficient mice [44].

Furthermore, dysferlin‐deficient mice exhibit escalated levels of immunoproteasome (IP) expression, typically associated with inflammatory responses entailing cytokine generation and antigen processing for presentation on MHC‐I. Administration of an IP inhibitor suppresses C3aR1 and C5aR1 while amplifying M2‐related signaling in dysferlin‐deficient mice, effectively restoring muscle function by curbing muscle infiltrations and fibro‐adipogenesis [45]. Inflammation‐associated alterations have also been documented in various other types of muscular dystrophies, including Duchenne muscular dystrophy (DMD), facioscapulohumeral dystrophy (FSHD), and calpainopathy [46, 47]. However, the deposition of diffuse myofiber complement C5b‐9 complex in muscle biopsies stands as a distinctive pathological hallmark of dysferlinopathy [48].

Our proteomics data have verified an upregulated expression of proteins linked to immune response and complement activation in dysferlinopathy‐afflicted muscles, aligning with the outcomes of a previous transcriptomics investigation [49]. Within this context, several proteins integral to the complement system, including C1, C3, C4B, C5‐6, and C8‐9, exhibited differential upregulation when compared to controls. We observed an elevation in the expression of three proteins (LBP, TRIM25, and ICAM1) associated with the NF‐kappaB pathway, indicating their involvement in the immune response to inflammatory challenges. Additionally, our study has identified an augmentation in complement C3 levels in dystrophic muscle, which correlated with pathological alterations in the muscle tissue, such as variability in fiber sizes, necrosis, and regeneration. The genetic elimination of complement C3 resulted in an enhanced muscle phenotype in dysferlin‐deficient mice, implying the potential efficacy of complement‐suppression therapies [50].

### 4.2 Annexins in Repair, Annexins A1, A2, A4, and A5 Expression Level Correlate with Muscle Histopathology

Annexins, particularly AnxA1, AnxA2, AnxA4, AnxA5, and AnxA6, play a crucial role in the process of plasma membrane repair (PMR). They facilitate membrane blebbing, promote the shedding of damaged membrane components, and contribute to the stability of the injured plasma membrane [51, 52, 53]. Both ANXA1 and ANXA2 can exist as monomers in the cytosol. However, upon binding to calcium ions through their N‐terminal domains, they can also form heterotetramers with S100 proteins, promoting the aggregation of intracellular vesicles and the formation of lipid rafts on the cytosolic surface of plasma membranes [54, 55, 56].

Upon membrane injury, the influx of calcium ions triggers the patch fusion process. Dysferlin interacts with Annexins A1 and A2, promoting the aggregation of intracellular vesicles and their fusion with the sarcolemma [14]. Unlike Annexins A1 and A2, AnxA4 and AnxA5 can autonomously assemble into trimers on membrane surfaces [57]. Subsequent plasma membrane injury and the influx of Ca^2+^, ANXA6 is recruited to the wound edges, where it initiates constriction of the hole edges. The translocation of ANXA6 and other annexins to the wound site facilitates the aggregation and fusion of endosomes and other vesicles with the damaged membrane [58]. Simultaneously, monomeric ANXA4 is attracted to the injured membrane, forming trimers and inducing local out‐of‐plane curvature [59]. Upon activation by Ca^2+^, AnxA5 binds to phosphatidylserine (PS)‐containing membranes, self‐assembling into 2D arrays on membranes, further facilitating membrane repair [60].

Microarray RNA analysis revealed an overexpression of annexin A2 in dysferlinopathy muscle compared with normal subjects [49]. A subsequent study further demonstrated elevated levels of annexins A1 and A2 in skeletal muscles affected by dysferlinopathy and other muscular disorders, indicating that annexin levels are not exclusive markers for dysferlinopathies. However, it was observed that annexin levels positively correlated with clinical severity and muscle histopathology in dysferlinopathy, suggesting their potential use as prognostic indicators of the disease [61]. Our proteomics data indicate that, in addition to annexins A1 and A2, increased expression levels of annexins A4 and A5 exhibit a significant positive correlation with muscle dystrophic pathology alterations. These findings suggest that multiple annexins contribute to the dystrophic process, which appears to be a secondary phenomenon in the progression of muscle disease. The upregulation of annexin proteins may be associated with an increase in Ca^2+^ concentration in muscle fibers in dysferlinopathy, where membrane repair is defective, potentially serving as compensation for plasma membrane injuries.

### 4.3 The Nuclear Envelope Protein Lamin (Isoforms A/C, B)

Our proteomic analyses unveiled a notable elevation in the levels of Lamin A/C and Lamin B1 in dysferlinopathy. However, we exclusively identified a significant association between the expression of Lamin A/C and the extent of dystrophic pathology. Prior investigations have similarly documented alterations in Lamin A/C protein levels in patients with dysferlinopathy, with an observed upregulation of Lamin A/C and B1 in dystrophic (mdx) mouse muscles, identifying them as dystrophic markers [21, 62]. Lamins, a class of nuclear intermediate filament proteins, assume responsibility for nuclear stability, and provide structural linkage between muscle cell nuclei and the cytoskeleton. The heightened presence of lamin isoforms A/C and B1 likely promotes the formation of fibrous structures through lamin proteins. Subsequently, this facilitates the preservation of structural integrity in the inner nuclear membrane during the phases of muscle degeneration and regeneration, ultimately imparting stability to muscle fibers affected by inflammatory processes.

### 4.4 Downregulation of Muscle Glycogen Phosphorylase, Perturbations in the Energy Metabolism of Dysferlinopathy

Glycogen, a polymer comprising glucose molecules, typically ranging from a few hundred to up to 55,000, forms through the linkage of α‐1,4‐glycosidic and α‐1,6‐glycosidic branching bonds. The initial step of glycogenolysis is catalyzed by glycogen phosphorylase (PYGM), releasing glucose‐1‐phosphate by breaking down the α‐1,4‐glycosidic bonds within the glycogen structure [63, 64].

In muscle tissue, the primary role of PYGM is to provide the necessary energy for the contraction of myofibrils. The glycogen debranching enzyme, AGL (amylo‐alpha‐1‐6‐glucosidase‐4‐alpha‐glucanotransferase), functions as both a glycosyl transferase and glucosidase during glycogen debranching [65, 66]. Our profiling results indicate the downregulation of PYGM and AGL expression, suggesting impaired glycogen metabolism in dysferlinopathy (Figure S2). Moreover, the decreased expression of PYGM is associated with the progression of muscle issues in dysferlinopathy. Glycerol‐3‐phosphate dehydrogenase 1 (GPD1) significantly influences both carbohydrate and lipid metabolism by facilitating the bidirectional transformation of dihydroxyacetone phosphate (DHAP) and reduced nicotinamide adenine dinucleotide (NADH) into glycerol 3‐phosphate (G3P) and NAD^+^. Cytosolic GPD1, in conjunction with its mitochondrial counterpart, constitutes the GPD1 shuttle, vital for the oxidation of glycolytic NADH within the mitochondria [67, 68, 69]. Our proteomic profiling reveals a notable decrease in cytoplasmic GPD1 levels, indicating a potential reduction in mitochondrial oxidation of NADH from the cytosol and its consequent impact on triglyceride metabolism in dysferlinopathy. This finding is particularly significant considering previous reports of lipid accumulation and elevated levels of triacylglycerol (TAG) in the muscles of dysferlin‐deficient mice [70].

Our proteomic study identified disruptions in the energy metabolism associated with dystrophic pathology. Notably, there was a significant downregulation of enzymes crucial for glycogen metabolism, including PYGM, GPD1, phosphoglucomutase‐1 (PGM1), and Triosephosphate isomerase (TPI1) (Figure S2). Despite the diminished levels of glycogen‐related proteins, we did not detect the accumulation of glycogen granules in dysferlinopathy muscle pathology. This observation suggests a potential impairment in both glycogenesis and glycogenolysis processes, indicating the complexity of glycogen metabolism regulation, necessitating further investigation.

In summary, our study conducted a thorough investigation into the proteomic profiles of muscle tissues affected by dysferlinopathy, demonstrating variations in the severity of dystrophic pathology. We identified significant associations between the altered expression levels of Annexins, LMNA and PYGM and the histopathology grading scale. This correlation holds the potential for development into a comprehensive prognostic biomarker signature, facilitating the monitoring of disease progression in dysferlinopathy studies. Our findings propose novel targets and biomarker candidates for the diagnostic development and therapeutic intervention of dysferlinopathy.

## Author Contributions

F.L. and Z.‐q.W.: developed the study concept and design. Q.‐F.H., F.‐z.Z., X.L., L.C., Y.Z., Y.‐h.L., M.‐h.Z., and H.‐z.C.: collected the clinical information. X.‐y.L., Q.‐F.H., M.‐t.L., and F.L.: preformed the human tissue isolation, processing, and histological analyses. F.L., D.W., M.‐t.L., Q.‐F.H., F.‐z.Z., N.W., and Z.‐q.W.: organized and analyzed the data. F.L. and D.W.: wrote the manuscript. Z.‐q.W. and F.L.: supervised the study. All authors have read and approved the final manuscript.

## Conflicts of Interest

The authors declare no conflicts of interest.

## Supporting information


**Figure S1.** Scatter plots showing the correlation of expression of selected proteins (X‐axis) and clinical characteristics (Y‐axis).

**Figure S2.** Heatmap of the expression of proteins related to energy metabolism.

## Data Availability

The data that support the findings of this study are available from the corresponding author upon reasonable request.
