# Supplementary figures and images for: Comprehensive Proteomic Analysis of Dysferlinopathy Unveiling Molecular Mechanisms and Biomarkers Linked to Pathological Progression
Source: CNS Neurosci Ther. 2024 Sep 30;30(10):e70065. doi: 10.1111/cns.70065 (PMC11442333; doi:10.1111/cns.70065)

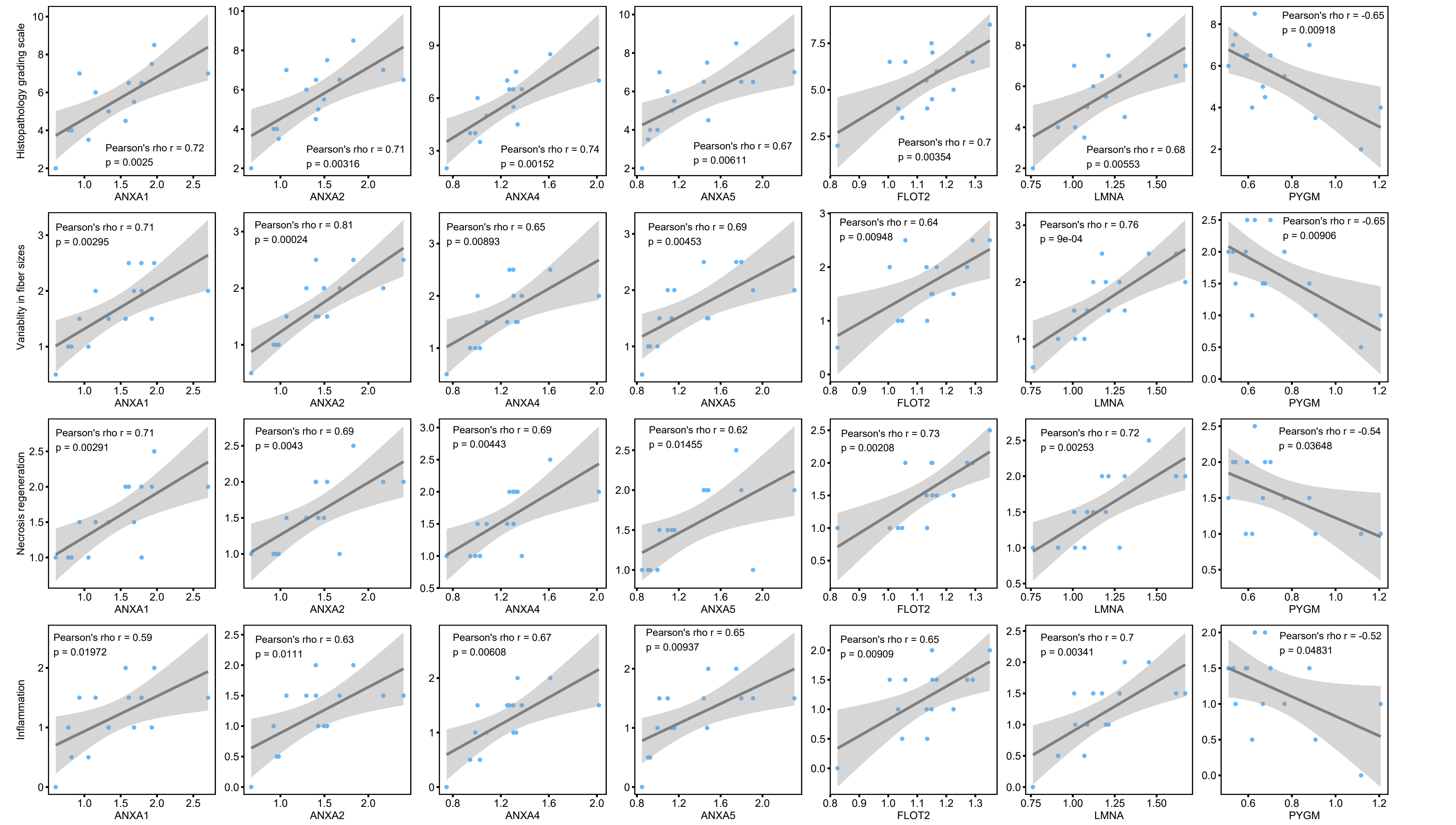

Supplement: Supplementary file 1 — Figure S1. Scatter plots showing the correlation of expression of selected proteins (X‐axis) and clinical characteristics (Y‐axis). [file CNS-30-e70065-s001.pdf]

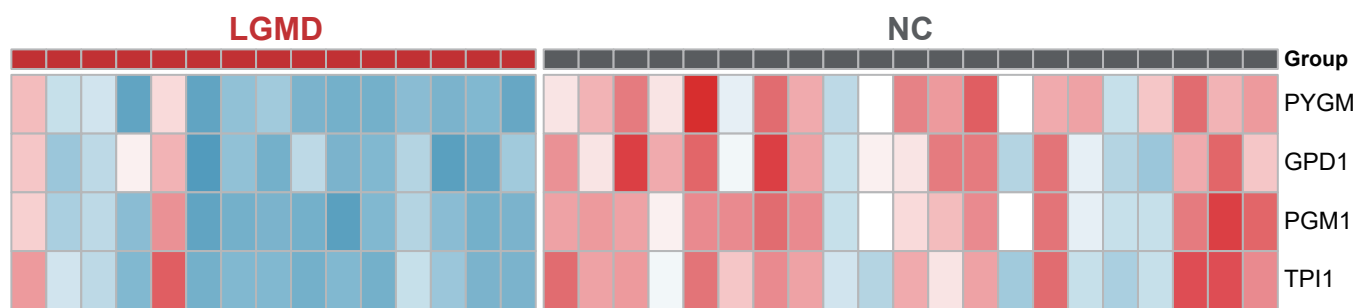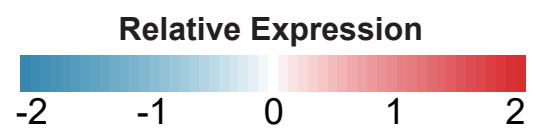

Supplement: Supplementary file 2 — Figure S2. Heatmap of the expression of proteins related to energy metabolism. [file CNS-30-e70065-s002.pdf]
